# Prevalence and Risk Factors of Anemia among Children 6–59 Months Old in Haiti

**DOI:** 10.1155/2013/502968

**Published:** 2013-03-10

**Authors:** Mohamed Ag Ayoya, Ismael Ngnie-Teta, Marie Nancy Séraphin, Aissa Mamadoultaibou, Ellen Boldon, Jean Ernst Saint-Fleur, Leslie Koo, Samuel Bernard

**Affiliations:** ^1^UNICEF Country Office, 125 Rue Faubert, Petionville, Port-au-Prince, Haiti; ^2^Saint Boniface Foundation, 12 Rue E. Guello, Fond des Blancs, Haiti

## Abstract

Anemia has serious consequences on child growth, development, and survival. This study was conducted in Fond des Blancs and Villa, Haiti, to assess the prevalence of childhood anemia and its risk factors in order to inform program design. Children 6–59 months old (*n* = 557) were selected using a cross-sectional multistage sampling methodology. Hemoglobin was measured using the HemoCue technique. Descriptive and multivariate analyses were performed to determine prevalence and factors associated with anemia. The prevalence of childhood anemia was 38.8% (23.9% mild, 14.7% moderate, and 0.2% severe). Mean hemoglobin was 11.2 ± 1.2 g/dL. Variables associated with child anemia were age less than 24 months (OR = 2.6; *P* = 0.000), stunting (OR = 2.2; *P* = 0.005), and mother's low hemoglobin level (OR = 1.8; *P* = 0.011). Anemia among young children in Fond des Blancs and Villa is a public health problem. Predictors of child anemia in this region include child's age, stunting, and mother's anemia. Interventions and strategies aimed at addressing effectively anemia in this population must therefore target mothers and children under two years of age.

## 1. Introduction

Childhood anemia is a major public health problem worldwide. It is associated with serious consequences including growth retardation, impaired motor and cognitive development, and increased morbidity and mortality [1]. Estimates suggest that 47.4% of children under five years of age are anemic globally [2]. In Haiti, the 2005-2006 nationally representative survey showed that 60.6% of children 6–59 months (approximately 610,000 children) and 75% of 6–23 months were anemic [3]. Data on childhood anemia and its predictors are lacking in Haiti. This hinders the design of programs and limits their effectiveness. This study was hence carried out to determine the prevalence and risk factors of childhood anemia in Fond des Blancs and Villa, a socioeconomically disadvantaged, remote, mountainous region where the Health Department, UNICEF, and Saint Boniface Foundation planned to implement a community-based mother and child nutrition program. Therefore, the study was designed to initiate new activities to prevent childhood anemia in this region in addition to iron and folic acid supplementation for pregnant women and vitamin A + albendazole for children 6–59 months. These new activities include intense promotion of optimal breastfeeding practices and hand washing with soap, large distribution of multiple micronutrient powders to children older than 6 months, improved availability of safe drinking water, and improved availability and utilization of latrines. 

## 2. Materials and Methods

### 2.1. Subjects

Children 6–59 months old (*n* = 557) were randomly selected using a two-stage random sampling scheme in 30 of the 69 villages of the area. The list of all villages and 2009 population estimates were used. The first sampling stage represents the selection of clusters, and the second sampling stage is the selection of households within the clusters. 

### 2.2. Study Site

The survey took place in Fond des Blancs and Villa, located in the South and South-East departments of Haiti, respectively. They are rural, poor, isolated, mountainous, and difficult-to-reach areas because of poor roads particularly during the rainy season. The population for the most part practice subsistence farming and raise goats and pigs as their main source of income. For over 27 years, Saint Boniface Haiti Foundation has been delivering education and community development programs in this region. The foundation also sponsors Saint Boniface Hospital, a 60-bed facility serving a large catchment area consisting of 120,000 people from four sections of the southern peninsula and one section from southeastern department. 

### 2.3. Measurements

Hemoglobin levels of children and the mother were measured using the HemoCue. Anemia was defined as hemoglobin <11 g/dL for children and pregnant women and hemoglobin <12 g/dL for nonpregnant women. Weight and height were measured for all children. Age was obtained from a birth certificate or a child health card. Weight-for-age (WAZ), height-for-age (HAZ), and weight-for-height (WHZ) *z*-scores were calculated using the World Health Organization Child Growth Standards Macro for SPSS [4]. Underweight, stunting, and wasting were defined as WAZ < −2.0, HAZ < −2.0, and WHZ < −2.0 standard deviations (SD) below the 2006 WHO reference, respectively. 

### 2.4. Statistical Analyses

Descriptive statistics were calculated. Means (±SD) were derived for hemoglobin, and random effects logistic regression modeling was used to determine the risk factors associated with childhood anemia. All analyses were performed using the Windows' SPSS version 20.0. 

### 2.5. Ethical Clearance, Informed Assent, and Consent

The study was approved by the health department and Saint Boniface Hospital authorities. Consents were obtained from children's parents before inclusion in the study. Treatment for anemia was provided to anemic subjects free of charge.

## 3. Results

**Figure 1:**
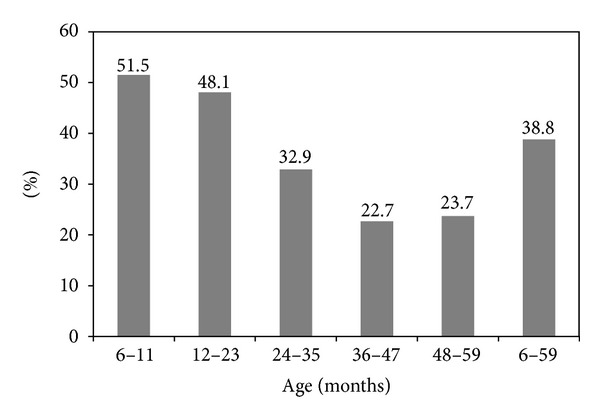
Prevalence of anemia among children by age group.

The boy : girl sex ratio was almost 1.0 (0.9). The prevalence of anemia among children was 38.8% (23.9% mild, 14.7% moderate, and 0.2% severe). Mean hemoglobin concentration was 11.2 ± 1.2 g/dL. The prevalence of anemia was slightly higher among boys (42.1%) than girls (35.7%).  Figure 1 shows the prevalence of anemia among children by age categories.

Among non-pregnant and pregnant women, the prevalence of anemia was 23.9% and 29.2%, and mean hemoglobin levels were 12.9 ± 1.5 g/dL and 11.8 ± 1.3 g/dL, respectively. 

The prevalence of child wasting was 4.5% (95% CI: 3.2–6.2) with 1.1% (95% CI: 0.5–2.4) being severe; 19.8% (95% CI: 16.5–23.6) of children were stunted and 9.8% (95% CI: 7.6–12.5) were underweight.

Variables associated with child anemia were age group under 24 months (OR = 2.6; 95% CI: 1.7–3.8, *P* = 0.000), stunting (OR = 2.2; 95% CI: 1.4–3.6; *P* = 0.005), and mother's low hemoglobin (OR = 1.8; 95% CI: 1.2–2.9; *P* = 0.011) (Table 1).

## 4. Discussion

This is the first population-based study to document childhood anemia prevalence and its risk factors in the region of Fond des Blancs and Villa and one of the very few in Haiti on this subject. We found that almost 4 children out of 10 are anemic and that boys are more affected than girls. Similar results were found in another study conducted in urban Haitian children two to five years old [5]. We also found that child's age, height-for-age *z* score < −2, and mother's anemia predict childhood anemia in this population. The same factors were found in other studies [6, 7]. The levels of anemia observed among young children and pregnant women and the high prevalence of stunting among under-five children in this study may also indicate chronic deprivations and hence a less favorable socioeconomic status of the population studied.

The prevalence of anemia among children 6–59 months in Fond des Blancs, though high, is still lower than that in the same age group nationally (60.6%) and in the south (54.4%) and southeastern (43.8%) departments where Fond des Blancs and Villa are located [3]. A plausible explanation for the better anemia indicators in this study could be the long presence of Saint Boniface in this region. Saint Boniface provides a variety of services including basic maternal and child health care services, behavior change communication, monthly supplemental dry rations for pregnant and lactating women, blanket feeding for children 6–23 months of age, and community-based distribution of micronutrients (vitamin A and iodine capsules) to young children and women. A study conducted in other departments of Haiti where similar services were provided to the population found that age-based preventive targeting of food assistance and behavior change communication reduced childhood undernutrition [8]. The limitation of this study is its cross-sectional nature. Its strengths are that it provides important information for policy makers, program planners, and implementers that seek to reduce childhood anemia in Haiti and that it was conducted in a poor and vulnerable population group. Our findings have important programmatic implications. First, they clearly show that the burden of anemia is on children under 24 months of age, a group in which the use of multiple micronutrient powders to reduce anemia has been shown in Haiti [9]. Second, they reinforce the current focus on the 1000 days, a window of opportunity that shapes the child's future and during which nutrition interventions offer a high return on investments. Third, they represent a powerful tool of advocacy to influence national policy and program actions and invest more in maternal and child nutrition.

## 5. Conclusion

Our findings showed that in this rural setting of Haiti, based on WHO criteria, anemia among young children is a moderate public health problem (prevalence between 20% and 40%). Factors to consider for reducing the burden of this disease on children comprise child's age, stunted growth, and hemoglobin status of the mother.

## Figures and Tables

**Table 1:** Risk factors associated with childhood anemia in Haiti.

Risk factors	OR	95% CI	*P* value
Age < 24 months	2.6	1.7–3.8	0.000
Sex = male	1.3	0.8–1.9	0.247
WAZ < −2	0.9	0.4–2.2	0.917
WHZ < −2	1.2	0.4–3.3	0.704
HAZ < −2	2.2	1.4–3.6	0.005
Mother's anemia	1.8	1.2–2.9	0.011

OR: odd ratios; CI: confidence interval; WAZ: weight-for-age *z* score; WHZ: weight-for-height *z* score; HAZ: height-for-age *z* score.
